# Layer-Specific Global Longitudinal Strain Predicts Arrhythmic Risk in Arrhythmogenic Cardiomyopathy

**DOI:** 10.3389/fcvm.2021.748003

**Published:** 2021-11-15

**Authors:** Diego Segura-Rodríguez, Francisco José Bermúdez-Jiménez, Lorena González-Camacho, Eduardo Moreno Escobar, Rocío García-Orta, Juan Emilio Alcalá-López, Alicia Bautista Pavés, José Manuel Oyonarte-Ramírez, Silvia López-Fernández, Miguel Álvarez, Luis Tercedor, Juan Jiménez-Jáimez

**Affiliations:** ^1^Cardiology Department, Hospital Universitario San Cecilio, Granada, Spain; ^2^Instituto de Investigación Biosanitaria ibs.GRANADA, Granada, Spain; ^3^Cardiology Department, Hospital Universitario Virgen de las Nieves, Granada, Spain; ^4^Centro Nacional de Investigaciones Cardiovasculares, CNIC, Instituto de Salud Carlos III, Madrid, Spain

**Keywords:** sudden cardiac death (SCD), late gadolinium enhanced, non-sustained ventricular tachycardia, arrhythmogenic cardiomyopathy (ACM), global longitudinal strain

## Abstract

**Background:** Arrhythmogenic cardiomyopathy (AC) is a life-threatening disease which predispose to malignant arrhythmias and sudden cardiac death (SCD) in the early stages of the disease. Risk stratification relies on the electrical, genetic, and imaging data. Our study aimed to investigate how myocardial deformation parameters may identify the subjects at risk of known predictors of major ventricular arrhythmias.

**Methods:** A cohort of 45 subjects with definite or borderline diagnosis of AC was characterized using the advanced transthoracic echocardiography (TTE) and cardiac magnetic resonance (CMR) and divided into the groups according to the potential arrhythmic risk markers, such as non-sustained ventricular tachycardia (NSVT), late gadolinium enhancement (LGE), and genetic status. Layer-specific global longitudinal strain (GLS) by TTE 2D speckle tracking was compared in patients with and without these arrhythmic risk markers.

**Results:** In this study, 23 (51.1%) patients were men with mean age of 43 ± 16 years. Next-generation sequencing identified a potential pathogenic mutation in 39 (86.7%) patients. Thirty-nine patients presented LGE (73.3%), mostly located at the subepicardial-to-mesocardial layers. A layer-specific-GLS analysis showed worse GLS values at the epicardial and mesocardial layers in the subjects with NSVT and LGE. The epicardial GLS values of −15.4 and −16.1% were the best cut-off values for identifying the individuals with NSVT and LGE, respectively, regardless of left ventricular ejection fraction (LVEF).

**Conclusions:** The layer-specific GLS assessment identified the subjects with high-risk arrhythmic features in AC, such as NSVT and LGE. An epicardial GLS may emerge as a potential instrument for detecting the subjects at risk of SCD in AC.

## Introduction

Arrhythmogenic cardiomyopathy (AC) is a genetically determined myocardial disease characterized by the progressive fibro-fatty myocardial replacement leading to heart failure and life-threatening arrhythmias in the early stages of the disease (1). It is a clinically heterogeneous disease due to its incomplete penetrance and variable expression. Therefore, there is no single gold standard for the diagnosis of AC and the diagnostic process is considered challenging. The diagnosis of AC is currently made on the consensus based revised 2010 Task Force criteria (TFC) (2), and updated in 2019 with the Padua criteria for left sided forms (3). The imaging criteria underlines the importance of cardiac imaging in AC with a great influence of cardiac magnetic resonance imaging (CMR). The detection of a regional wall motion abnormality is required to score a major or minor criterion regardless of the outflow tract dilatation or systolic dysfunction. Moreover, the presence and location of late gadolinium enhancement (LGE) on CMR are reported as the predictive markers of ventricular arrhythmias and sudden cardiac death (SCD) (4, 5).

Through the post-mortem studies, it is established that fibrofatty replacement begins at the epicardium level with progressive extension to mesocardial layers (6). This fibrofatty involvement can be assessed non-invasively by using CMR through the LGE sequences allowing not only detection of scars, but also evaluate the location, extension, and distribution, which helps arrhythmic risk stratification and facilitates the selection of susceptible individuals at risk of developing malignant events (7).

Despite the CMR advances, an echocardiography remains a non-invasive, relatively inexpensive, widely available first-line diagnostic tool. The recent developments in echocardiography, as tissue imaging deformation (TID) mainly assessed by the speckle-tracking technology, may increase the performance of conventional echocardiography. Ejection fraction (EF) is a well-known classic echocardiographic parameter which describes the capacity of the ventricle to oust a determined volume, which is a global systolic parameter and usually remains preserved when either the few segments are affected or at early stages of the disease (8). However, TID allows the regional wall performance analysis and detecting incipient pathological changes when the traditional echocardiography measures (i.e., volumes and EF) are still normal. Myocardial strain has proved as a wide clinical utility throughout numerous cardiovascular areas: cardio-oncology, ischemic cardiomyopathy, valvular heart disease, and several non-ischemic cardiomyopathies, such as amyloidosis (9–12). The TID usefulness remains not only on its capability to detect the subclinical damage, but also to guide the medical and interventional treatment as well as to stratify the short- and long-term prognosis (13–15). Furthermore, the growing TID evidence has emerged in AC, demonstrating the diagnostic and prognostic value which may guide decision-making for arrhythmic primary prevention (16, 17).

Since arrhythmic events and SCD may occur in the absence of a definite diagnosis, there is a need to identify new tools to facilitate the earliest diagnosis and risk stratification. Some authors have attempted to assess the value of speckle-tracking strain in the early diagnosis and disease progression of AC (18). However, there is lack of information on the arrhythmic prognostic value of speckle-tracking strain. This study aimed to evaluate the association of TID with the major SCD risk factors as non-sustained ventricular arrhythmia, fibrous scar on CMR, or genetic background.

## Methods

### Study Population and Clinical Evaluation

We retrospectively recruited 45 subjects with definite or borderline diagnosis of AC, based on 2010 TFC, who underwent transthoracic echocardiography and CMR (2). The patients were evaluated between 2007 and 2020 at the Inherited Cardiomyopathies Unit of two tertiary hospitals. The study was approved by the Institutional Review Board and the Local Ethics Committee, and all the participants signed the informed consent. We excluded all the patients with permanent pacemaker pacing, ischemic heart disease, more than mild valvular involvement (stenosis/regurgitation), and poorly controlled hypertension.

The clinical assessment comprised exhaustive evaluation of medical history, family history of SCD or cardiomyopathy, 12-lead electrocardiogram, basic laboratory test, and genetic testing. In addition, 24-h Holter monitoring, echocardiography, and CMR were obtained within 6 months for each patient. We thoroughly assessed the medical history for arrhythmic events: (a) non-sustained ventricular (NSVT) tachycardia defined as ≥3 consecutive premature complexes with a heart rate of >120 beats/min lasting <30 s, and (b) a composite of (1) ventricular tachycardia/ventricular fibrillation (VT/VF) defined as the presence of a ventricular rhythm at a rate >120 beats/min that lasts longer than 30 s, (2) the incidence aborted cardiac arrest due to VF which is reversed by the successful resuscitation maneuvers, and (3) the incidence of an appropriate implantable cardioverter-defibrillator (ICD) shock when they occurred in response to VT or VF.

The peripheral blood samples for the genetic analysis were obtained from the probands or the deceased index case, as applicable. A next-generation sequencing (NGS) gene panel containing 21 genes (previously associated with the development of arrhythmogenic cardiomyopathy) was applied (Supplementary Material). The pathogenicity of the identified variants was classified according to the current guidelines of the American College of Medical Genetics and Genomics (ACMG) (19). After a potential disease-causing variant was identified in the index patient, the genetic and clinical cascade were conducted. The subjects were classified according to the genetic test results as the desmosomal mutations carriers, non-desmosomal carriers, and negative/unknown mutations carriers.

### Cardiovascular Imaging Analysis

The echocardiography and CMR imaging acquisition, interpretation and analysis were performed by the two experienced, independent, and blinded imaging specialists. The acquisition protocols and post-processing are described in the Supplementary Material.

#### Echocardiography

Transthoracic echocardiography acquisition was performed using Vivid 9 system (GE® Healthcare, Hørten, Norway). The size of chamber, quantifications, and severity partition cut-offs of left ventricular (LV) dysfunction were measured according to the current guidelines (20). We used an 18-segment model to analyze the regional wall motion abnormalities (RWMA) and deformation assessment. The images were acquired at 65 frames per second and processed offline using Echo-PAC software GE® (GE®, Hørten, Norway).

The strain analyses were performed using a dedicated software (EchoPac strain package for analysis, GE® Healthcare, Hørten, Norway), tracing endocardial border, and adjusting region of interest avoiding pericardium. We obtained the global longitudinal strain (GLS) by using a 2D speckle-tracking method (21). We evaluated: layer-specific GLS, regional longitudinal strain, and mechanical dispersion from the analysis of the SD (MD_SD_) and range between maximum and minimum time value (MD_delta_) of the time to reach peak negative strain. The GLS-specific and mechanical dispersion values were analyzed according to the references values (22–24). We calculated the ratio of endocardial GLS to epicardial GLS (Endo-Epi GLS ratio) using the endocardial GLS/epicardial GLS for the assessment of the strain gradient, as previously described (23). Twenty patients were randomly selected for analyzing interobserver variability by another observer blinded to the results of the first reader, assessing the GLS at each myocardial layer (GLS_epi_, GLS_meso_, and GLS_endo_).

#### CMR Study

All the patients underwent a CMR evaluation. The LV and right ventricular (RV) function were categorized according to the current guidelines (25). We considered the presence of LV involvement when any of the following conditions were present: LV RWMA, LV wall thinning, left ventricular ejection fraction (LVEF) <50%, or LGE with non-ischemic pattern. On the other hand, right ventricular (RV) involvement was considered according to TFC (2). The LGE sequences were qualitatively assessed (presence, location, and layer-distribution) according to an 18-segment model.

Finally, the patients were classified on the basis of RV and/or LV involvement as follows: lone RV (isolated RV involvement), biventricular, LV dominant (isolated LV involvement), and negative CMR (absence of any CMR signs).

### Statistical Analysis

The qualitative variables were described using the absolute frequencies and percentages. The continuous variables were expressed as mean and SD, or median, when applicable. The normality of the data was tested with Shapiro–Wilk test.

A comparative analysis between the groups was performed using Pearson's chi-square test or Fisher's exact test for the qualitative variables. Intergroup comparisons for the quantitative variables were made using Student's *t*-test or Mann–Whitney *U*-test when indicated. For quantitative comparison among the three groups, an ANOVA test or Kruskal–Wallis were performed. Interobserver variability was evaluated using the intraclass correlation coefficient (ICC) for layer-specific GLS analysis. The receiver operating characteristics (ROC) curves were used to define the GLS cut-offs able to predict the NSVT and LGE. A value *p* < 0.05 was considered statistically significant. The data were processed using the SPSS Statistics 25 software (IBM®, Armonk, NY, USA).

## Results

**Figure 1:**
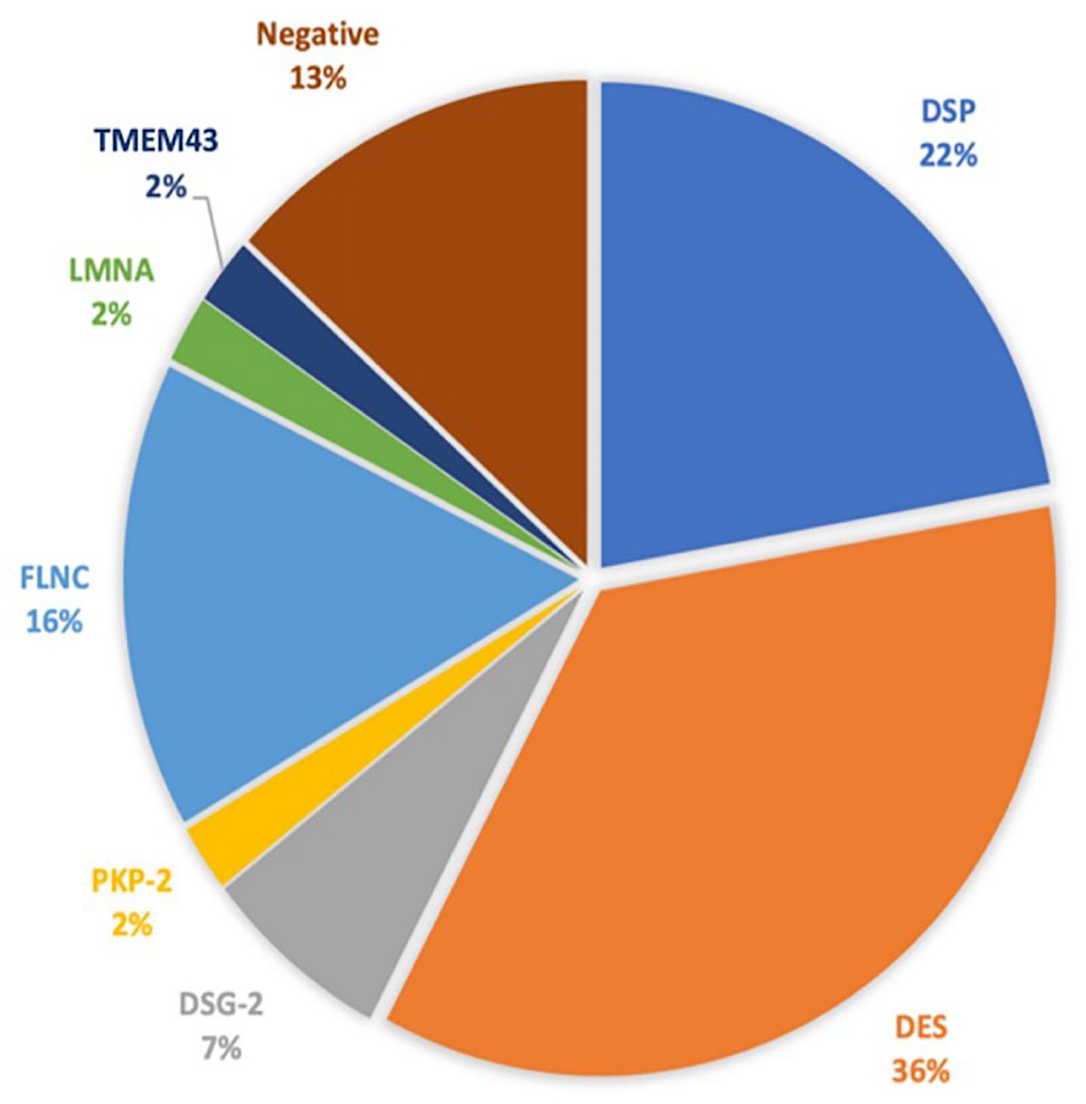
The genetic test results. DSP, desmoplakin; DES, desmin; DSG-2, desmoglein-2; PKP-2, plakophilin; FLNC, filamin C; LMNA, lamin A/C; TMEM43, transmembrane protein 43.

The baseline clinical characteristics are summarized in Table 1. We included 37 Caucasian patients with a definite and eight patients with a borderline diagnosis of AC (mean age 43 ± 16 years and 51% were men), belonging to the 19 families, who had previously undergone a TTE and CMR. Overall, a high prevalence of family history of SCD was found (39; 86.7%). Twenty-four patients (53.3%) had cardiac symptoms at first evaluation, mainly dyspnea (10; 22.2%) and palpitations (7; 15.6%). The asymptomatic patients were diagnosed primarily by family cascade screening. In 37 patients, we found a pathogenic or likely a pathogenic variant (Figure 1 and Supplementary Table 1). During 24 h Holter monitoring or 12-lead ECG, we identified 16 patients (35.5%) presenting NSVT. The incidence of NSVT was not significantly associated with LVEF (51.9 ± 8.3 vs. 47.7 ± 10.7%; *p* = 0.15) or indexed LV end-diastolic volumes (55.5 ± 15 vs. 58.8 ± 17 ml/m^2^; *p* = 0.497).

**Table 1:** The baseline characteristics.

	**Overall (45)**	**VT/VF (-) (33)**	**VT/VF (+) (12)**	***p*-value**
Sex (male) *n*, (%)	23 (51.1)	12 (36.4)	11 (91.1)	0.001
Age (years), mean ± SD	43.13 ± 16.5	41.3 ± 17.8	48.3 ± 11.6	0.21
Body mass index, kg/m^2^	26.8 ± 4.1	27.2 ± 4.4	25.8 ± 3.4	0.31
Body surface area, m^2^	1.8 ± 0.2	1.83 ± 0.2	1.96 ± 0.21	0.08
Hypertension *n*, (%)	2 (4.4)	2 (6.1)	0 (0)	1
Dyslipidaemia *n*, (%)	2 (4.4)	1 (3)	1 (8.3)	0.46
Type 2 diabetes *n*, (%)	1 (2.2)	1 (3)	0 (0)	1
Tobacco use				0.66
Active smoking *n*, (%)	4 (8.9)	3 (9.1)	1 (8.3)	
Former smoking *n*, (%)	2 (4.4)	2 (6.1)	0 (0)	
Family history SCD, *n* (%)	39 (86.7)	33 (100)	6 (50)	<0.001
Asymptomatic, *n* (%)	21 (46.7)	20 (60.6)	1 (8.3)	0.002
Symptoms, *n* (%)	24 (53.3)			<0.001
Dyspnoea	10 (22.2)	8 (24.2)	2 (16.7)	
Chest pain	2 (4.4)	2 (6.1)	0 (0)	
Palpitations	7 (15.6)	1 (3)	6 (50)	
Syncope	3 (6.7)	2 (6.1)	1 (8.3)	
Aborted SCD	2 (4.4)	0 (0)	2 (16.7)	
NYHA class I *n*, (%)	34 (75.6)	24 (72.7)	10 (83.3)	0.021
NYHA class II *n*, (%)	9 (20)	9 (27.3)	0 (0)	
NYHA class III *n*, (%)	2 (4.4)	0 (0)	2 (16.7)	
Abnormal ECG *n*, (%)	34 (75.6)	23 (69.7)	11 (91.7)	0.24
Atrial fibrillation *n*, (%)	4 (8.9)	2 (6.1)	2 (16.7)	0.28
Paroxysmal	2 (4.4)	1 (50)	1 (50)	
Persistent	1 (2.2)	0	1 (50)	
Permanent	1 (2.2)	1 (50)	0	
Genetic testing *n*, (%)				<0.001
Desmosomal mutation	15 (33.3)	11 (33.3)	4 (16.7)	
Non-desmosomal mutation	24 (53.3)	22 (66.7)	2 (16.7)	
Unknown	6 (13.3)	0	6 (50)	
Phenotype,^†^ *n* (%)				0.022
RV	5 (11.1)	2 (6.1)	3 (25)	
LV	19 (42.2)	17 (51.1)	1 (8.3)	
BV	15 (33.3)	9 (27.3)	7 (58.3)	
Silent	6 (13.3)	5 (15.2)	1 (8.3)	
NSVT *n*, (%)	16 (35.6)	7 (21.2)	9 75)	0.002
ICD *n*, (%)	23 (51.1)	13 (39.4)	10 (83.3)	0.001
Primary prevention *n*, (%)	14 (60)	12 (92.3)	2 (20)	
Secondary prevention *n*, (%)	9 (40)	1 (7.7)	8 (80)	
ICD shocks *n*, (%)	7 (15)	2 (6.1)	5 (41.7)	0.011
Appropriate *n*, (%)	5 (71.4)	0 (0)	5 100	
Inappropriate *n*, (%)	2 (28.6)	2 (100)	0 (0)	
LGE presence *n*, (%)	33 (73.3)	23 (69.7)	10 (83.3)	0.46
LGE phenotype *n*, (%)				0.297
RV:	10 (22.2)	8 (24.2)	2 (16.7)	
LV:	5 (11.1)	2 (6.1)	3 (25)	
BV:	18 (40)	13 (39.4)	5 (41.7)	

*CMR, cardiac magnetic resonance; Cardiac SCD, sudden cardiac death; RV, right ventricle; LGE, late gadolinium enhancement; LV, Left Ventricle; BV, Biventricular; ICD, Implantable cardioverter defibrillator*.

† *Based on the regional wall motion abnormalities and late gadolinium enhancement*.

Twelve patients (26.6%) had experienced the composite outcome of VT/VF with five individuals receiving an appropriate ICD shock.

An echocardiographic analysis revealed that nearly half of the patients (23; 51.1%) showed an impaired LVEF (mean 42.9 ± 5.9%), with 69.6% of the patients presenting with mildly reduced, 26.1% moderately reduced, and 4.3% severely reduced LVEF. In addition, majority of the patients had no RV systolic dysfunction [mean tricuspid annular plane systolic excursion (TAPSE) 20.1 ± 4 mm]. Overall, the structural evaluation showed a normal LV end-diastolic volume (LVEDV 104.9 ± 28.9 ml and indexed LVEDV 56.7 ± 15.6 ml/m^2^). Moreover, 31 patients presented regional RWMA at evaluation, mainly located at basal (23; 51.1%) and mid inferolateral (24; 53.3%) segments and mid lateral segments (23; 51.1%). In all the individuals, it was feasible to evaluate regional and GLS according to each myocardial layer. The GLS analysis results are shown in Supplementary Table 2. The mechanical dispersion parameters were MD_SD_ 51.3 ± 18 ms and MD_delta_ 174.2 ± 61.5 ms, being higher values than the normal ranges in the healthy population (24).

Regarding the AC appearance on CMR, a nearly exclusive LV involvement was the most frequent AC phenotype (42.2%), with 15 (33.3%) presenting a biventricular (BV) affection, and 5 (11.1%) a predominant RV involvement. The LV and RV ejection fraction (RVEF) distributions are displayed at Supplementary Figures 1, 2. The mean LVEF and RVEF values were 51.7 ± 10.2 and 50.9 ± 10.1%, respectively. With respect to LGE, 33 (73.3%) patients presented LGE with non-ischemic pattern. Distribution of LGE was identified as biventricular in 18 patients (54.5%), lone LV in 10 subjects (30.3%), and lone RV in five cases (15.2%). LV-LGE was predominantly located at the subepicardial-to-mesocardial layer (*n* = 27; 96.4%) and only one patient had focal patchy transmural LGE at the interventricular septum. None of the patients had subendocardial LGE involvement. In addition, LV-LGE was predominantly located at the lateral/inferolateral wall in 27 individuals (96.4%), with an extended circumferential pattern in 19 (70.37%) of these patients.

Among the patients who experienced VT/VF during follow-up, the BV phenotype was the most frequently encountered (7, 58.3%), followed by the exclusive RV (3, 25%) and LV phenotype (1, 8.3%).

### GLS and Major Arrhythmic Events

The layer-specific GLS comparing the patients with and without previous VT/VF [VT/VF (+) vs. VT/VF (–)] showed in the epicardial GLS values differences (−14.4 ± 2.2 vs. −16.1 ± 3.1%; *p* = 0.09), with no differences at either mesocardial or endocardial GLS analysis.

### Strain Association With Arrhythmic Risk Markers

#### GLS and NSVT

**Figure 2:**
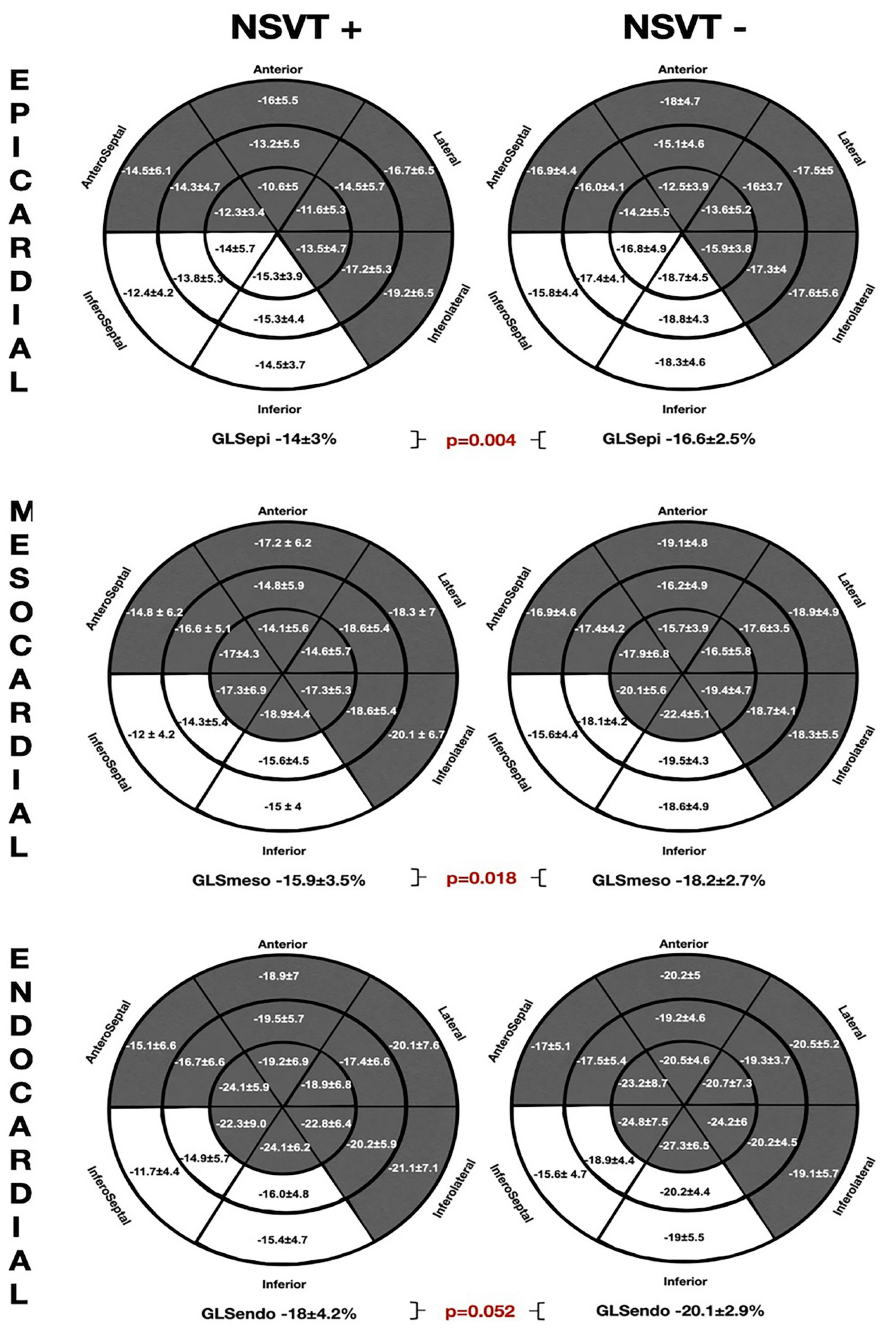
The comparative global and regional layer-specific GLS analysis global between the NVST (+) and NSVT (–) groups represented in the left ventricular (LV) 18-segment models according to each myocardial layer. White areas remark significant differences in regional longitudinal strain between the groups. GLS, global longitudinal strain; NSVT, non-sustained ventricular tachycardia.

The comparative regional and GLS analysis according to myocardial layer between the patients with history of NSVT [NSVT(+)] and without NSVT [NSVT(–)] is shown in Figure 2. The LV layer-specific GLS analysis showed poorer LV-GLS values in the NSVT(+) group at the mesocardial and epicardial layers. However, no significant differences were found between the groups when comparing GLS at the endocardial myocardial layer. Similarly, the mechanical dispersion parameters were not different in both the groups [MD_SD_: NSVT (+) 53.6 ± 15.6 ms vs. NSVT (–) 50 ± 19.3 ms; *p* = 0.434 and MD_delta_: NSVT (+) 185.6 ± 66.8 ms vs. NSVT (–) 167.9 ± 58.6 ms; *p* = 0.448].

Furthermore, the regional longitudinal strain at the inferoseptal and inferior basal-to-mid segments were consistently impaired within all the myocardial layers in the NSVT (+) group when compared with the NSVT (–) group. In addition, the Endo-Epi GLS ratio showed a tendency to the lower values in the NSVT (+) group (1.2 ± 0.1 vs. 1.3 ± 0.1; *p* = 0.06).

**Figure 3:**
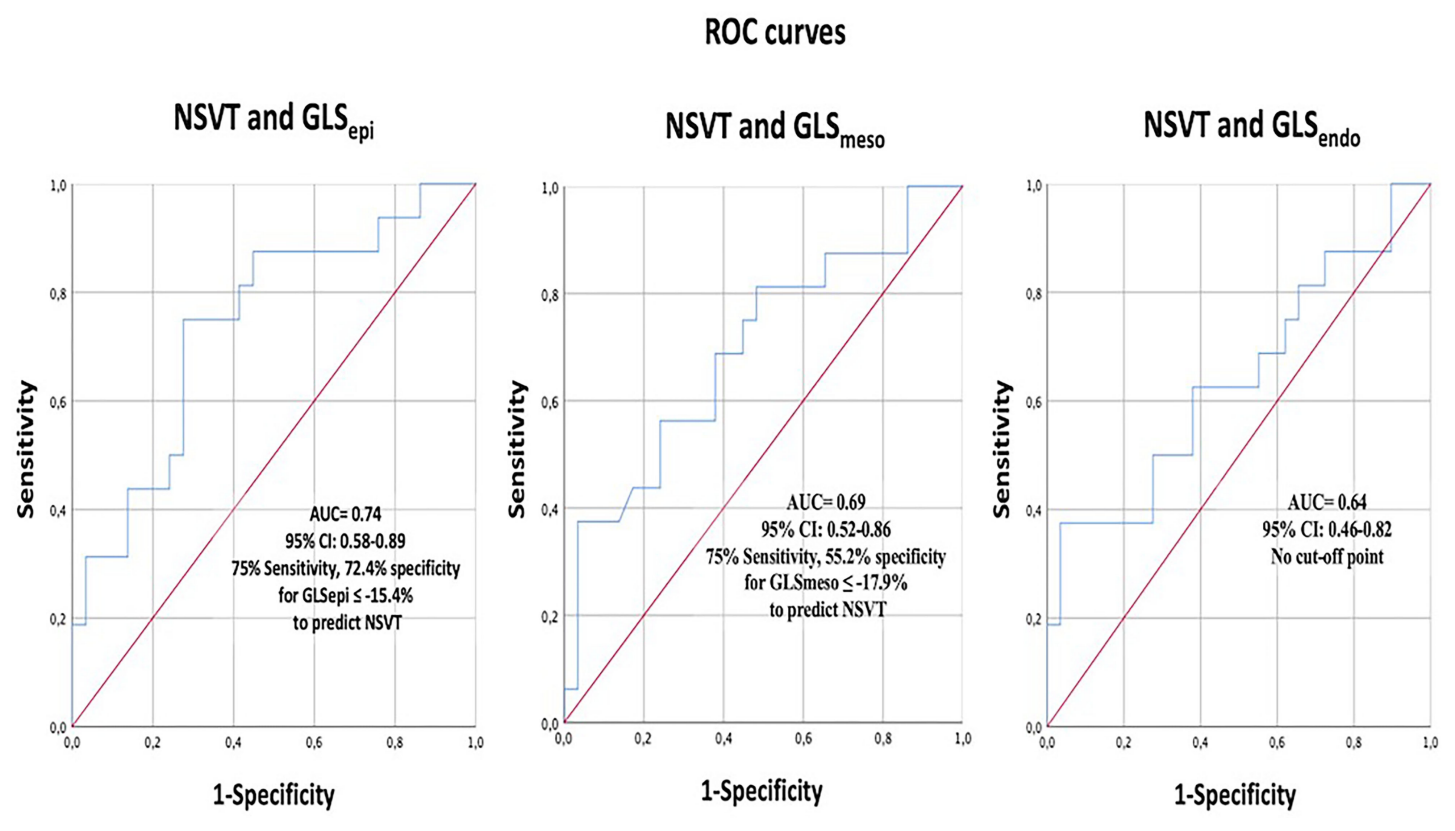
The receiver operating characteristics (ROC) curve analysis of layer-specific GLS to predict NSVT. GLS, global longitudinal strain; NSVT, non-sustained ventricular tachycardia.

In Figure 3, the ROC curves analysis of the layer GLS analysis to predict the presence of NSVT is represented. The best area under the curve (AUC) was 0.739 (95% *CI* 0.585–0.893) for GLS_epi_ with the best cut-off value −15.41%, giving a sensitivity of 75% and specificity of 72.4%.

#### GLS and LGE

**Figure 4:**
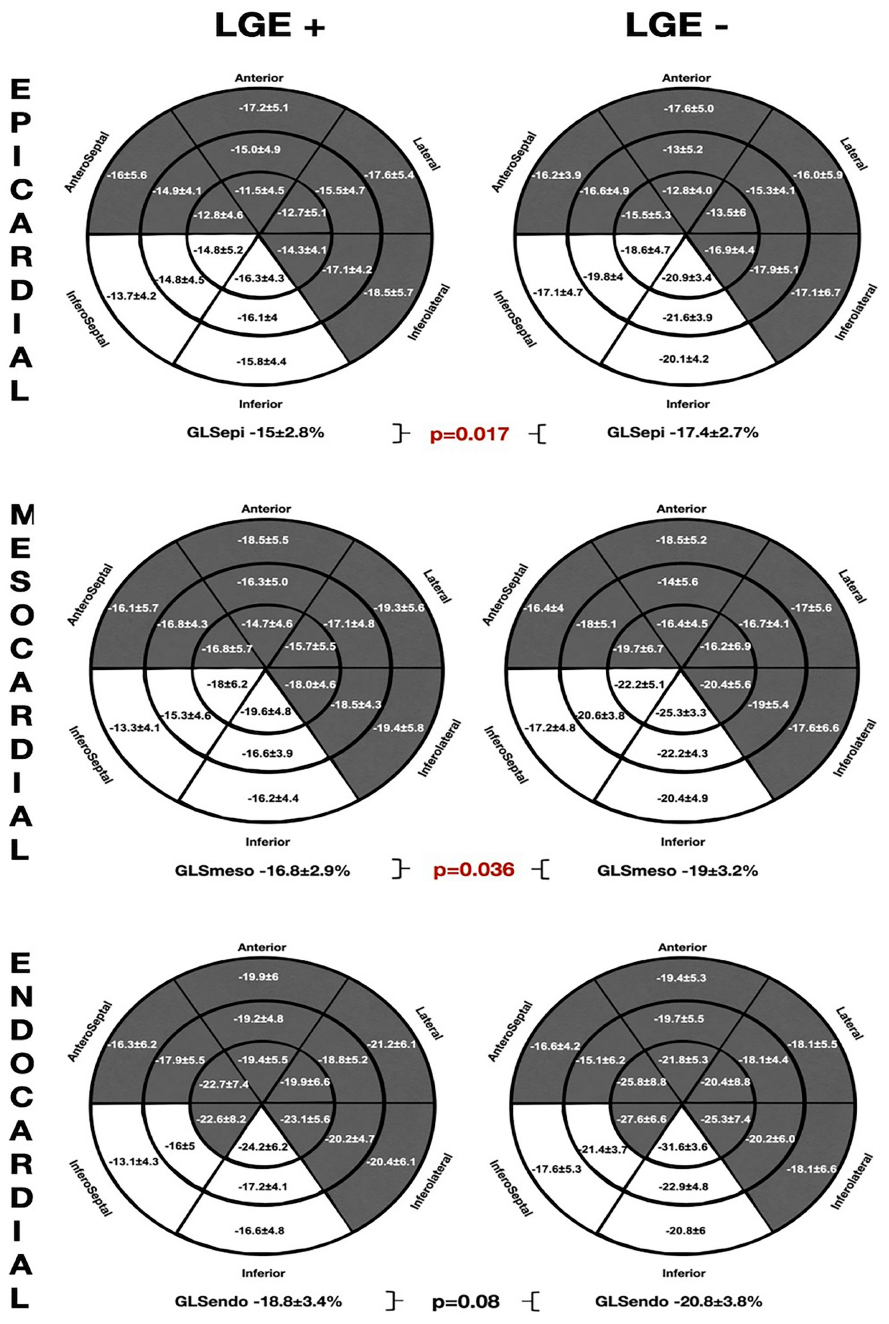
The comparative global and regional layer-specific GLS analysis global between the LGE (+) and LGE (–) groups represented in the LV 18-segment models according to each myocardial layer. White areas remark significant differences in regional longitudinal strain between the groups. GLS, global longitudinal strain; LGE, late gadolinium enhancement.

The comparative analysis between the layer-specific GLS and LGE presence (LGE+ vs. LGE–) is exposed at Figure 4. We found significant differences in GLS according to the LGE presence, specifically at the mesocardial and the epicardial layers. Conversely, there were no significant alterations in the endocardial layer GLS between the groups of patients with and without LGE. In addition, the Endo-Epi GLS ratio was lower in the LGE (+) group (1.3 ± 0.1 vs. 1.2 ± 0.06; *p* = 0.035).

As observed in the NSVT (+) group, the patients presenting LGE showed worse GLS values in the inferior and inferoseptal segments throughout all the myocardial layers. However, no significant differences were found on the mechanical dispersion parameters [MD_delta_: LGE (+) 52.2 ± 16.3 ms vs. LGE (–) 48.7 ± 22.7 ms; *p* = 0.57 and MD_delta_: LGE (+) 176.3 ± 57.1 ms vs. LGE (–) 168.5 ± 74.9 ms *p* = 0.36].

**Figure 5:**
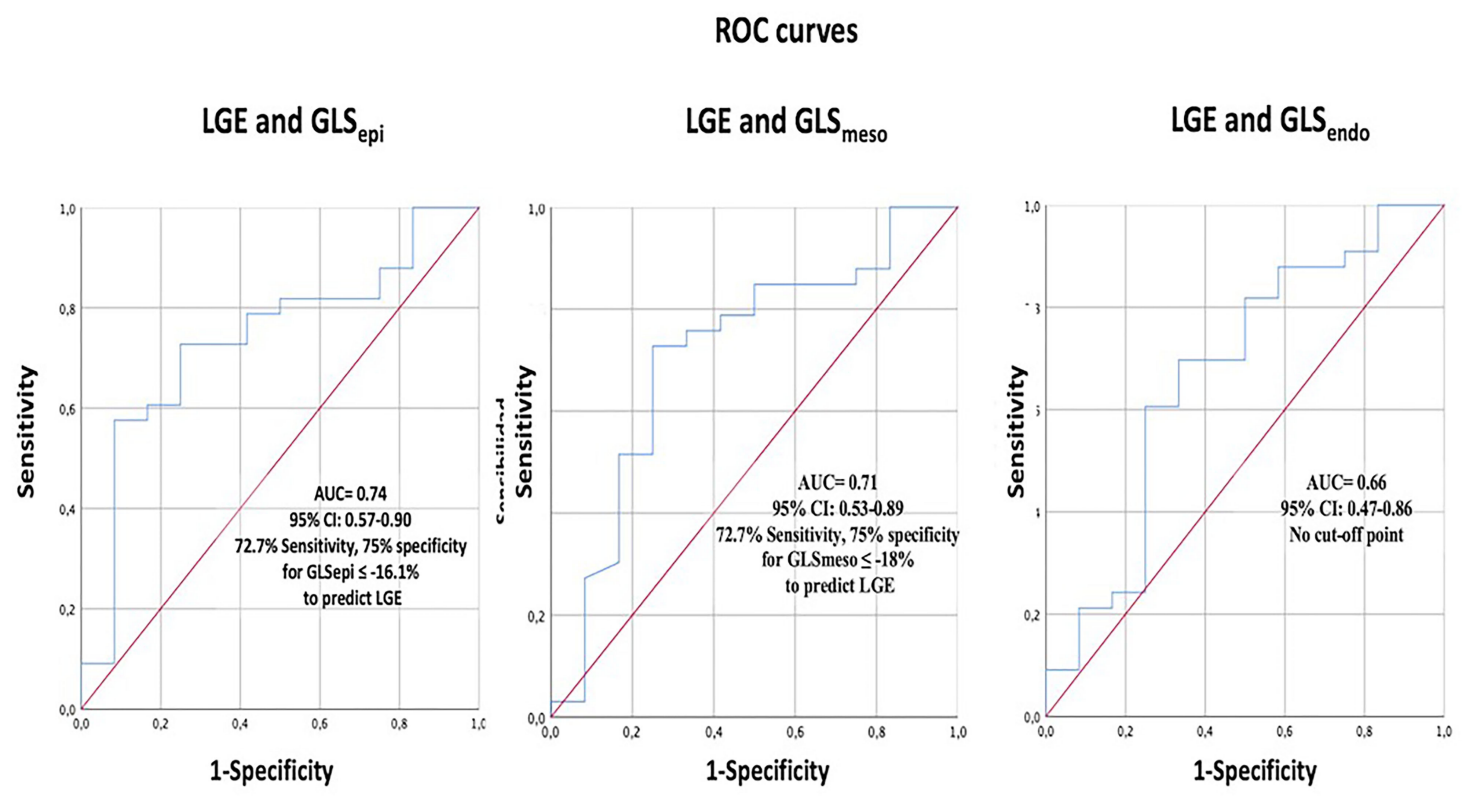
The ROC curve analysis of layer-specific GLS to predict the presence of LGE. GLS, global longitudinal strain; LGE, Late gadolinium enhancement.

Figure 5 depicts the ROC curves analysis of the layer GLS analysis to predict the presence of LGE. The best AUC was 0.75 (95% *CI* 0.0.57–0.9) for GLS_epi_ with the best cut-off value −16.1%, giving a sensitivity of 72.7% and specificity of 75%.

#### GLS and Genetics

We did not find significant differences in the GLS, dispersion parameters, or regional longitudinal strain analysis between the different genetic background (Supplementary Tables 3–5). Likewise, no significant differences were detected regarding the mechanical dispersion parameters (MD_SD_: desmosomal 52.2 ± 14.6 ms vs. non-desmosomal 50 ± 21.4 ms vs. unknown mutations 53.9 ± 11.6 ms; *p* = 0.89, MD_delta_: desmosomal 165.9 ± 37 ms vs. non-desmosomal 170.2 ± 68.2 ms vs. unknown mutations 211 ± 78.4 ms; *p* = 0.41) or Endo-Epi GLS ratio (desmosomal 1.3 ± 0.1 vs. non-desmosomal 1.2 ± 0.1 vs. unknown mutations *p* = 0.17).

#### Interobserver Analysis

An interobserver analysis showed excellent agreement between the observers with ICC 0.93 (95% *CI*, 0.80–0.97) for GLSepi, 0.93 (95% *CI*, 0.83–0.97) for GLSmeso and 0.87 (95% *CI*, 0.72–0.95) for GLSendo.

## Discussion

Arrhythmic risk stratification in AC remains a matter of debate and can be challenging, particularly in the early stages of the disease. LVEF is a poor predictor with a remarkable incidence of SCD in the patients with preserved or mildly impaired systolic function, particularly in the certain genotypes, such as *FLNC, LMNA, TMEM43*, or *DES* (26–29). New stratification tools are needed, and advanced cardiac imaging is gaining relevance in this field. Our work is the first to correlate the LV regional layer-specific GLS analysis with the traditionally accepted arrhythmic risk factors of ventricular arrhythmias in AC, such as NSVT, LGE, or genetic status. Our results seem promising as we were able to detect, in a small cohort of the patients with AC, significant TID disturbances that were associated with the presence of classical SCD risk factors, such as the presence of LGE or NSVT.

In general, LVEF remains one of the cornerstones during the decision-making process to select the high-risk patients who may benefit from the ICD implantation for primary prevention (19, 20). However, the LVEF loses discriminative capability in the setting of AC, as ventricular arrhythmias (VAs) or SCD might happen during the so-called electrical phase in the subjects with no evident macroscopic structural changes and normal LVEF. Therefore, it is mandatory to detect more sensitive parameters capable of detecting the pathological changes in the vulnerable phase before LVEF impairment. The present study aimed to go beyond the LVEF and standard average GLS, performing a layer-specific analysis to detect the subjects with the stablished arrhythmic risk markers. Several studies have shown that greater GLS (more positive) and LV mechanical dispersion might be the markers of ventricular arrhythmias but to date have not been included in the solid predictive models (16).

Nonetheless, NSVT and LGE are the well-recognized risk factors for arrhythmic risk. NSVT is classically included in the prediction models algorithms for VAs in AC (30–33). More recently, the LGE has shown remarkable prognostic value in AC showing that LV involvement and LV dominant phenotype are the independent factors of major events (34). Hence, searching these elements during the patient evaluation is of upmost relevance. In this sense, our study has shown that especially epicardial GLS had a good association for detecting the subjects with NSVT or LGE, pointing the best cut-off at ~-16 to −15%. Adding epicardial GLS to the routinely echocardiographic assessment may increase the sensitive capability to detect the subjects potentially at risk of VAs and therefore, of SCD.

The previous studies have shown that LV dominant AC presents a typical subepicardial-to-mesocardial LGE distribution, with a specific distribution at the inferolateral and lateral walls with circumferential extension (ring-like patterns) (35). Majority of our cohort of patients had subepicardial-to-mesocardial LV-LGE distribution and only one subject had patchy mesocardial fibrosis located at the interventricular septum. A good correlation observed, not only between the LGE and impaired GLS, but also in the distribution of the segments, reinforcing the potential of speckle-tracking TTE as a useful tool to detect the individuals with early involvement and as an arrhythmic risk predictor.

It has been previously described that the endocardial GLS is higher than epicardial GLS in the normal subjects with a Endo-Epi GLS ratio of ~1.3 (36). This might be explained by the differences in wall stress (more stress in the endocardial fibers during diastole making them larger than epicardial fibers) or changes in coronary perfusion (37, 38). Nevertheless, this Endo-Epi GLS gradient is likely accentuated when the progressive fibrosis accumulation occurs in the epicardial layers which is the central pathophysiology of AC. Indeed, lateral LV epicardium is affected before endocardium in AC (18). In our cohort, only at the epicardial and mesocardial level, the differences were observed between the patients at higher and lower arrhythmic risk, as defined by the presence of arrhythmic risk markers.

Despite of the potential clinical usefulness of TID by *speckle tracking*, it is mandatory to always check the tracking results visually and defining a good region of interest tracing (excluding pericardium and blood pool). When performed by expertise personnel, GLS has shown higher accuracy than the conventional diagnostic echocardiographic parameters, providing both the higher sensitivity and specificity to detect AC (39, 40).

However, due to the small sample size of this study, assumption in the terms of prognosis needs to be evaluated in larger prospective studies, taking into account major events, such as ventricular fibrillation, sustained ventricular tachycardia, or sudden cardiac death. In this regard, the integrating parameters, such as family history, LGE, arrhythmic burden, and genetics and potentially, the layer-specific GLS, such as epicardial GLS may increase the predictive capability to select the high-risk individuals who may benefit for the ICD implantation.

## Limitations

These data should be interpreted with caution due to the small sample size. In addition, the retrospective and cross-sectional design nature of this study does not allow inferences in the terms of prognosis. Hence, the VT events were included only through either Holter monitoring or 12-lead ECG which may have lower yield when compared with the monitoring using an implanted cardiac device. Furthermore, we are aware of the inherent limitations in ejection fraction, GLS, and LGE interpretation. Definition of region of interest in the speckle-tracking analysis is of utmost importance as it requires accurate tracing to avoid pericardial inclusion which may cause differential bias (21). The values of longitudinal strain may vary depending on age, sex, and vendor specific software, so that these values may not be applicable in a different population. Since major arrhythmic events were not used as the primary endpoint because there were few in such relatively rare pathology, future prospective, larger, and multicenter studies are needed to evaluate the predictive capacity of GLS to detect individuals at risk of malignant arrhythmic events need to be confirmed in a prospective study.

## Conclusion

The layer-specific GLS assessment identified the subjects with high-risk arrhythmic markers, such as NSVT and LGE presence. An epicardial GLS analysis showed the best of the ability for detecting the subjects with the arrhythmic risk factors. The larger prospective studies may correlate layer-specific GLS evaluation with the malignant arrhythmic events and its prognostic role.

## Data Availability Statement

The raw data supporting the conclusions of this article will be made available by the authors, without undue reservation.

## Ethics Statement

The studies involving human participants were reviewed and approved by Comité Ético de Investigación Clínica (CEI) de la provincia de Granada. The patients/participants provided their written informed consent to participate in this study.

## Author Contributions

DS-R: conceptualization, methodology, statistical analysis, investigation, writing the original draft, images elaboration, and coordination. FB-J and JJ-J: conceptualization, methodology, supervision, writing-review and editing, and clinical perspectives. LG-C, EM, RG-O, JO-R, and AB: validation, clinical evaluation, and image acquisition. JA-L and SL-F: imaging interpretation and analysis. MÁ and LT: clinical perspectives and discussion. All authors reviewed and approved the final manuscript.

## Conflict of Interest

The authors declare that the research was conducted in the absence of any commercial or financial relationships that could be construed as a potential conflict of interest.

## Publisher's Note

All claims expressed in this article are solely those of the authors and do not necessarily represent those of their affiliated organizations, or those of the publisher, the editors and the reviewers. Any product that may be evaluated in this article, or claim that may be made by its manufacturer, is not guaranteed or endorsed by the publisher.
