# Supplementary material for: Layer-Specific Global Longitudinal Strain Predicts Arrhythmic Risk in Arrhythmogenic Cardiomyopathy
Source: Front Cardiovasc Med. 2021 Nov 15;8:748003. doi: 10.3389/fcvm.2021.748003 (PMC8634435; doi:10.3389/fcvm.2021.748003)
Supplement: Supplementary file 1 [file Data_Sheet_1.docx]

**SUPPLEMENTARY MATERIAL**

**SUPPLEMENTARY METHODS**

The echocardiography study protocol consisted of the following views: left ventricle (LV) parasternal long axis, parasternal short axis (at mitral valve level, papillary muscles and apex), apical 4 chambers view, focused-dedicated LV views (4 chambers, 3 chambers and 2 chambers views) acquired at 65 frames per second at least. Additional images were acquired when required. LV volumes and LVEF by biplane Simpson method were also analysed.

CMR study included scout images (axial, sagittal, and coronal), double inversion recovery pulse sequence (dark blood images), structure and function module with balanced steady-state free precession (SSFP) sequence (short axis, LV-four-chamber, LV-three-chamber, LV-two-chamber, and RV-two chamber and RV outflow tract). Finally, 0.1–0.2 mmol/kg of chelated gadolinium (*Gadovist*®) were administered, obtaining the LGE images using an inversion recovery sequence 7–10min post-injection. Some additional sequences were acquired where clinically indicated. Post-processing analysis was performed using semi-automatic software (*Reportcard*®). LGE sequences were qualitatively assessed (presence, location and layer-distribution) according to an 18-segment model.

All the patients gave their informed consent for the administration of gadolinium and underwent a CMR, which was performed on a 1.5-T MR. system (General Electric, Signa EXCITE ®, Milwaukee, WI, USA).

**SUPPLEMENTARY FIGURE 1**


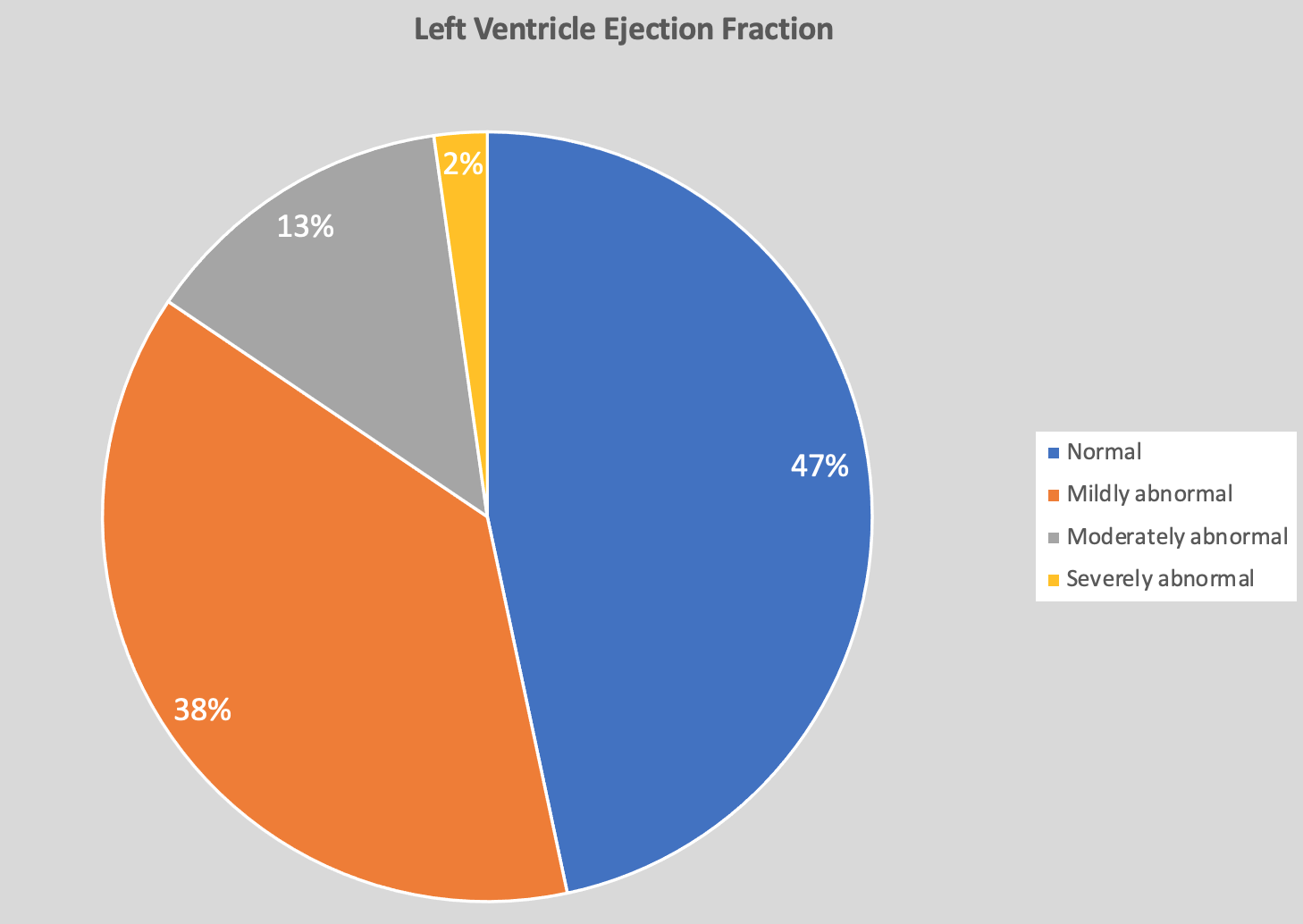


Supplementary Figure 1. Left Ventricular ejection fraction distribution according to Cardiac Magnetic Resonance.

**SUPPLEMENTARY FIGURE 2**


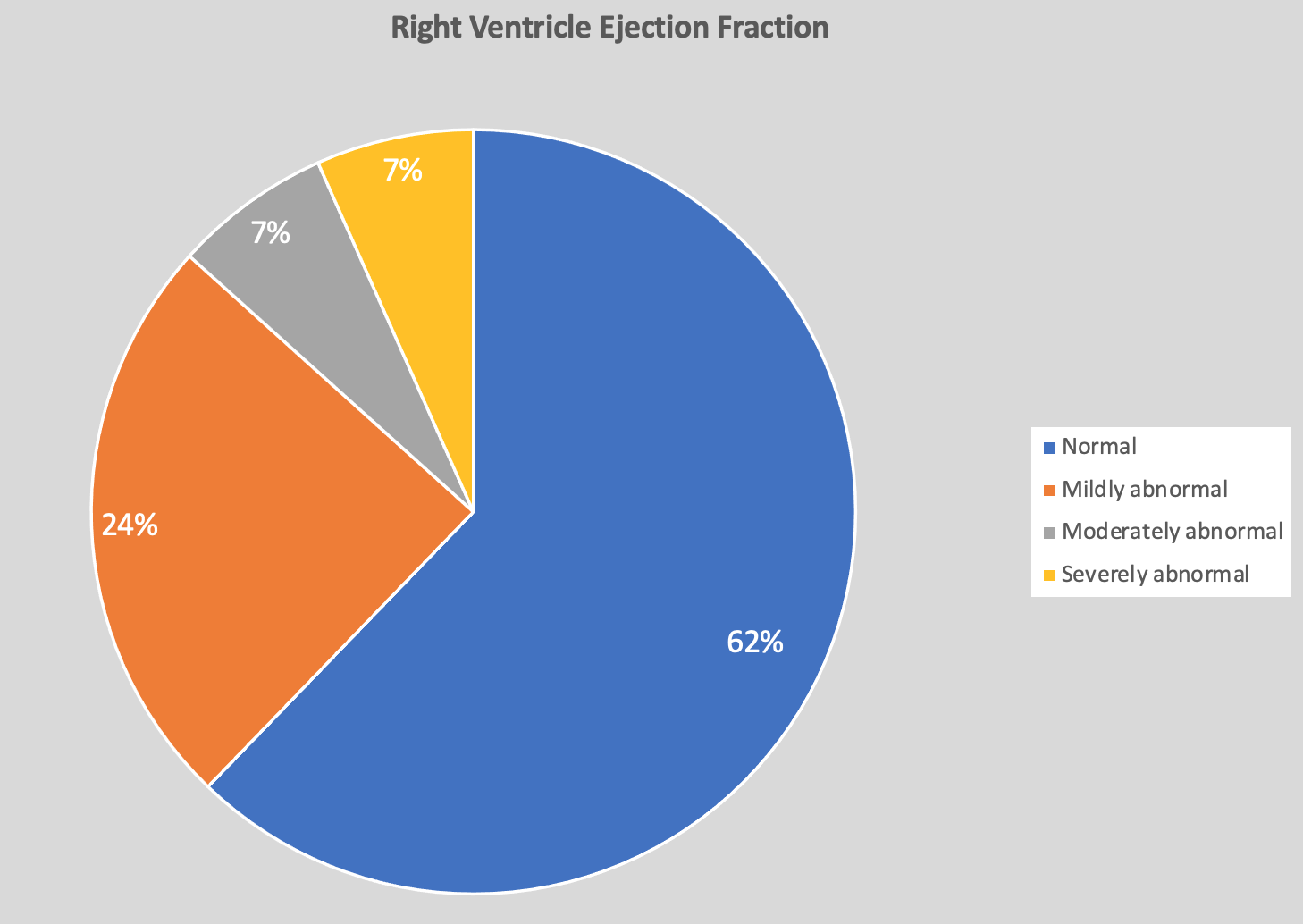


Supplementary Figure 2. Right Ventricular ejection fraction distribution according to Cardiac Magnetic Resonance.

**SUPPLEMENTARY TABLE 1**

| Gene | Protein | p-notation | c-notation | Carrier status | Variant type |
| --- | --- | --- | --- | --- | --- |
| *DES* | Desmin | p.Glu401Asp | c.1203G>C | Heterozygous | Missense |
|  |  | p.Asp212Ala | c.641A>C | Heterozygous | Missense |
| *FLNC* | FilaminC | p.Leu194Profs*52 | c.581_599delTGGTGG | Heterozygous | Nonsense |
|  |  | p.? | c.4288+2T>G | Heterozygous | Nonsense |
| *DSP* | Desmoplakin | p.Arg1045* | c.3133C>T | Heterozygous | Nonsense |
|  |  | p.? | c.1266+1G>A | Heterozygous | Nonsense |
|  |  | p.Val2567Cysfs*14 | c.7697_7698insG | Heterozygous | Nonsense |
|  |  | p.Arg2284* | c.6850C>T | Heterozygous | Nonsense |
|  |  | p.Arg160* | c.478C>T | Heterozygous | Nonsense |
| *PKP2* | placophilin2 | p.Gly548Valfs*15) | c.1643delG | Heterozygous | Nonsense |
| *DSG2* | Desmoglein-2 | p.Arg292His | c.875G>A | Homozygous | Missense |
|  |  | p.Arg49His | c.146G>A | Heterozygous | Missense |
| *LMNA* | LaminA/C | p.Trp514* | c.1541G>A | Heterozygous | Nonsense |
| *TMEM43* | Transmembrane protein 43 | p.Ser358Leu | c.1073C>T | Heterozygous | Missense |

Supplementary Table 1. List of genetic variants identified among arrhythmogenic cardiomyopathy patients.

**SUPPLEMENTARY TABLE 2**

| Layer-specific LS analysis | Segment | Epicardial, % | Mesocardial, % | Endocardial, % |
| --- | --- | --- | --- | --- |
| Basal | Anterior | -17.3±5 | -18.5±5.3 | -19.8±5.8 |
|  | Anteroseptal | -16.1±5.1 | -16.2±5.3 | -16.4±5.7 |
|  | Inferoseptal | -14.6±4.6 | -14.3±4.6 | -14.3±4.9 |
|  | Inferior | -17±4.7 | -17.3±4.9 | -17.7±5.4 |
|  | Inferolateral | -18.2±5.9 | -18.9±6 | -19.8±6.3 |
|  | Lateral | -17.2±5.5 | -18.7±5.7 | -20.4±6.1 |
| Mid | Anterior | -14.4±5 | -15.7±5.2 | -19.3±4.9 |
|  | Anteroseptal | -15.4±4.3 | -17.1±4.5 | -17.2±5.7 |
|  | Inferoseptal | -16.1±4.8 | -16.7±5 | -17.5±5.2 |
|  | Inferior | -17.6±4.6 | -18.1±4.7 | -18.7±4.9 |
|  | Inferolateral | -17.3±4.4 | -18.6±4.6 | -20.2±5 |
|  | Lateral | -15.5±4.5 | -17±4.6 | -18.6±4.9 |
| Apical | Anterior | -11.8±4.4 | -15.2±4.6 | -20.1±5.5 |
|  | Anteroseptal | -13.5±4.9 | -17.6±6 | -23.5±7.8 |
|  | Inferoseptal | -15.8±5.3 | -19.1±6.2 | -23.9±8 |
|  | Inferior | -17.5±4.5 | -21.1±5.1 | -26.2±6.5 |
|  | Inferolateral | -15±4.3 | -18.7±5 | -23.7±6.1 |
|  | Lateral | -12.9±5.3 | -15.8±5.8 | -20±7.1 |
| GLS | — | -15.6±2.9 | -17.3±3.1 | -19.3±3.6 |

Supplementary Table 2. Regional and Global Longitudinal Strain analysis according to each myocardial layer. LS = Longitudinal Strain. GLS = global longitudinal strain.

**SUPPLEMENTARY TABLE 3**

| GLS Mesocardial n. (%) | Segment | Desmosomal  15, (33.3) | Non-desmosomal  24, (53.3) | Negative  6, (13.3) | p value |
| --- | --- | --- | --- | --- | --- |
| Basal | Anterior | -18.4±3.6 | -19.4±5.4 | -14.9±7.7 | 0.18 |
|  | Anteroseptal | -16.4±5.7 | -16.5±5.3 | -14.2±4.5 | 0.63 |
|  | Inferoseptal | -13.8±4.4 | -14.4±4.9 | -15.5±4.6 | 0.74 |
|  | Inferior | -18.1±5.4 | -16.9±5.0 | -17.0±2.7 | 0.74 |
|  | Inferolateral | -19.0±5.2 | -18.9±6.0 | -18.8±8.5 | 1.00 |
|  | Lateral | -19.5±4.2 | -18.8±6.2 | -16.2±6.9 | 0.49 |
| Mid | Anterior | -17.2±5.0 | -15.9±4.8 | -11.3±6.0 | 0.06 |
|  | Anteroseptal | -17.0±3.8 | -17.3±5.1 | -17.0±4.3 | 0.98 |
|  | Inferoseptal | -16.1±4.9 | -16.8±5.3 | -18.2±4.3 | 0.70 |
|  | Inferior | -17.6±4.4 | -18.4±5.3 | -18.0±3.5 | 0.89 |
|  | Inferolateral | -18.7±3.8 | -18.9±4.6 | -17.8±6.4 | 0.88 |
|  | Lateral | -18.1±3.9 | -17.0±4.9 | -14.4±4.5 | 0.26 |
| Apical | Anterior | -16.4±5.0 | -14.7±4.4 | -13.8±4.0 | 0.42 |
|  | Anteroseptal | -19.1±7.0 | -16.4±5.5 | -18.3±4.8 | 0.37 |
|  | Inferoseptal | -20.3±7.3 | -18.8±5.4 | -17.3±6.8 | 0.58 |
|  | Inferior | -20.6±5.6 | -21.3±5.2 | -21.6±4.3 | 0.9 |
|  | Inferolateral | -19.6±4.9 | -18.2±4.8 | -18.2±6.2 | 0.68 |
|  | Lateral | -18.2±4.8 | -15.5±5.9 | -11.4±5.9 | 0.08 |
| GLSmeso (%) | — | -17.7±3.1 | -17.3±3.3 | -16.6±2.9 | 0.79 |

Supplementary Table 3. Comparative Mesocardial global and regional Longitudinal Strain analysis among genetic groups. GLS = global longitudinal strain

**SUPPLEMENTARY TABLE 4**

| GLS Epicardial n, (%) | Segment | Desmosomal  15, (33.3) | Non-desmosomal 24, (53.3) | Negative  6, (13.3) | p value |
| --- | --- | --- | --- | --- | --- |
| Basal | Anterior | -17.2±3.3 | -18.3±5.2 | -13.5±6.6 | 0.11 |
|  | Anteroseptal | -16.2±5.4 | -16.6±5.2 | -13.8±4.2 | 0.52 |
|  | Inferoseptal | -14.4±4.6 | -14.5±4.5 | -15.3±5.4 | 0.91 |
|  | Inferior | -17.9±4.9 | -16.5±5.0 | -16.4±2.6 | 0.64 |
|  | Inferolateral | -18.0±5.1 | -18.3±5.8 | -17.9±9.1 | 0.98 |
|  | Lateral | -18.1±4.3 | -17.1±5.9 | -15.0±6.9 | 0.51 |
| Mid | Anterior | -16.1±4.8 | -14.6±4.5 | -9.9±5.1 | 0.03 |
|  | Anteroseptal | -15.0±4.0 | -15.7±4.7 | -14.9±4.0 | 0.85 |
|  | Inferoseptal | -15.6±4.4 | -16.1±5.3 | -17.5±4.1 | 0.71 |
|  | Inferior | -17.0±4.2 | -17.9±5.3 | -17.7±3.4 | 0.86 |
|  | Inferolateral | -16.7±3.2 | -17.7±4.6 | -17.0±6.6 | 0.81 |
|  | Lateral | -16.6±4.3 | -15.4±4.6 | -13.2±4.6 | 0.30 |
| Apical | Anterior | -13.0±4.8 | -11.7±4.3 | -9.5±3.3 | 0.26 |
|  | Anteroseptal | -14.6±5.9 | -12.9±4.4 | -13.4±4.2 | 0.59 |
|  | Inferoseptal | -16.6±6.3 | -15.7±4.7 | -13.9±5.3 | 0.57 |
|  | Inferior | -17.0±4.8 | -18.0±4.7 | -17.0±3.6 | 0.67 |
|  | Inferolateral | -15.5±4.3 | -14.8±4.1 | -14.5±5.6 | 0.86 |
|  | Lateral | -14.7±4.5 | -12.8±5.1 | -8.6±5.7 | 0.065 |
| GLSepi (%) | — | -15.8±2.8 | -15.8±3.2 | -14.7±2.5 | 0.7 |

Supplementary Table 4. Comparative Epicardial global and regional Longitudinal Strain analysis among genetic groups. GLS = global longitudinal strain.

**SUPPLEMENTARY TABLE 5**

| GLS Endocardial  n, (%) | Segment | Desmosomal  15, (33.3) | Non-desmosomal  24, (53.3) | Negative  6, (13.3) | p value |
| --- | --- | --- | --- | --- | --- |
| Basal | Anterior | -19.7±4.1 | -20.6±5.7 | -16.5±9.0 | 0.30 |
|  | Anteroseptal | -16.6±6.2 | -16.6±5.7 | -14.8±4.7 | 0.77 |
|  | Inferoseptal | -13.3±4.4 | -14.5±5.5 | -15.8±3.8 | 0.56 |
|  | Inferior | -18.3±6.0 | -17.4±5.6 | -17.6±3.5 | 0.87 |
|  | Inferolateral | -20.2±5.7 | -19.6±6.4 | -19.7±8.0 | 0.95 |
|  | Lateral | -20.9±4.3 | -20.7±6.8 | -17.5±6.9 | 0.46 |
| Mid | Anterior | -19.4±3.7 | -19.2±5.8 | -19.6±4.8 | 0.99 |
|  | Anteroseptal | -18.5±5.5 | -17.4±5.3 | -12.9±7.2 | 0.12 |
|  | Inferoseptal | -16.8±5.5 | -17.6±5.3 | -18.8±4.7 | 0.71 |
|  | Inferior | -18.3±4.9 | -19.0±5.4 | -18.4±3.8 | 0.91 |
|  | Inferolateral | -20.8±4.8 | -20.2±4.9 | -18.7±6.2 | 0.70 |
|  | Lateral | -19.7±3.9 | -18.7±5.4 | -15.8±4.5 | 0.26 |
| Apical | Anterior | -21.5±5.9 | -19.2±5.2 | -20.1±5.6 | 0.44 |
|  | Anteroseptal | -25.7±8.5 | -21.6±7.5 | -25.7±5.8 | 0.21 |
|  | Inferoseptal | -25.7±8.9 | -23.2±7.3 | -22.4±9.2 | 0.58 |
|  | Inferior | -26.0±6.9 | -25.7±6.4 | -28.3±6.7 | 0.70 |
|  | Inferolateral | -25.3±5.9 | -22.8±6.1 | -23.6±6.9 | 0.47 |
|  | Lateral | -23.1±5.7 | -19.2±7.5 | -15.8±6.8 | 0.07 |
| GLSendo (%) | — | -20.0±3.3 | -19.1±3.7 | -18.5±3.8 | 0.62 |

Supplementary Table 5. Comparative Endocardial global and regional Longitudinal Strain analysis among genetic groups. GLS = global longitudinal strain.
